# Lectures on Auto-Intoxication in Disease

**Published:** 1895-03

**Authors:** 


*Lectures on Auto-Intoxication in Disease*. By CH. BOUCHARD. Translated by THOMAS OLIVER, M.D. Pp. xvi., 302. Philadelphia: THE F. A. DAVIS COMPANY. London: F. J. REBMAN. 1894.

For many years Prof. Bouchard has been making experiments on the poisonous properties of the excretions in health and disease, and in this volume summarises his results, pointing out the part played by these poisons—which are produced both by the katabolism of the tissues and by putrefaction in the intestinal canal,—and giving the indications for treatment suggested by the investigation of their occurrence and properties.

The complexity of the subject is great: it is, for instance, somewhat bewildering to be told that normal urine contains seven toxic substances—an antipyretic, an organic and an inorganic convulsive substance, a diuretic, a myotic, a narcotic, and a sialogogue substance; so that failure in the excretion of one or more of these brings about the corresponding result. Thus, in uræmia it is found that the urine has no toxic properties, and that the varying nature of the symptoms—whether mainly convulsive or mainly comatose—depends on the proportions of the various poisons which are retained. Again, Prof. Bouchard shows that the bile is poisonous chiefly by reason of its pigments, and that as long as the kidneys excrete these there is no danger; whereas if they fail, the urine ceases to be toxic and symptoms of poisoning—cholæmia and uræmia—occur.

We owe Dr. Oliver a debt of gratitude for translating these lectures, which not only point out how many methods of treatment, which have been long practised, do good, but also are an important contribution to a deeper knowledge of the complex chemistry of the animal body and a corresponding increase in our power in the prevention and treatment of many diseases.

